# Indole, a Signaling Molecule Produced by the Gut Microbiota, Negatively Impacts Emotional Behaviors in Rats

**DOI:** 10.3389/fnins.2018.00216

**Published:** 2018-04-09

**Authors:** Mathilde Jaglin, Moez Rhimi, Catherine Philippe, Nicolas Pons, Aurélia Bruneau, Bénédicte Goustard, Valérie Daugé, Emmanuelle Maguin, Laurent Naudon, Sylvie Rabot

**Affiliations:** ^1^Micalis Institute, Institut National de la Recherche Agronomique, AgroParisTech, Université Paris-Saclay, Jouy-en-Josas, France; ^2^MetaGenoPolis, Institut National de la Recherche Agronomique, Université Paris-Saclay, Jouy-en-Josas, France; ^3^Micalis Institute, Institut National de la Recherche Agronomique, AgroParisTech, Centre National de la Recherche Scientifique, Université Paris-Saclay, Jouy-en-Josas, France

**Keywords:** microbiota, tryptophanase, indole, oxindoles, vagus nerve, behavior, anxiety, rat

## Abstract

Gut microbiota produces a wide and diverse array of metabolites that are an integral part of the host metabolome. The emergence of the gut microbiome-brain axis concept has prompted investigations on the role of gut microbiota dysbioses in the pathophysiology of brain diseases. Specifically, the search for microbe-related metabolomic signatures in human patients and animal models of psychiatric disorders has pointed out the importance of the microbial metabolism of aromatic amino acids. Here, we investigated the effect of indole on brain and behavior in rats. Indole is produced by gut microbiota from tryptophan, through the tryptophanase enzyme encoded by the *tna*A gene. First, we mimicked an acute and high overproduction of indole by injecting this compound in the cecum of conventional rats. This treatment led to a dramatic decrease of motor activity. The neurodepressant oxidized derivatives of indole, oxindole and isatin, accumulated in the brain. In addition, increase in eye blinking frequency and in c-Fos protein expression in the dorsal vagal complex denoted a vagus nerve activation. Second, we mimicked a chronic and moderate overproduction of indole by colonizing germ-free rats with the indole-producing bacterial species *Escherichia coli*. We compared emotional behaviors of these rats with those of germ-free rats colonized with a genetically-engineered counterpart strain unable to produce indole. Rats overproducing indole displayed higher helplessness in the tail suspension test, and enhanced anxiety-like behavior in the novelty, elevated plus maze and open-field tests. Vagus nerve activation was suggested by an increase in eye blinking frequency. However, unlike the conventional rats dosed with a high amount of indole, the motor activity was not altered and neither oxindole nor isatin could be detected in the brain. Further studies are required for a comprehensive understanding of the mechanisms supporting indole effects on emotional behaviors. As our findings suggest that people whose gut microbiota is highly prone to produce indole could be more likely to develop anxiety and mood disorders, we addressed the issue of the inter-individual variability of indole producing potential in humans. An *in silico* investigation of metagenomic data focused on the *tna*A gene products definitively proved this inter-individual variability.

## Introduction

The human gut is inhabited by a microbial ecosystem whose collective genome encodes 500 times more genes than the human genome (Li et al., 2014). The combination of this huge genetic potential with the myriad substrates supplied by dietary nutrients and by the gastro-intestinal and hepatic secretions results in the production of a wide and diverse array of metabolites, whose influence on the host systemic metabolome is considerable. Comparing the serum metabolome of germ-free and conventional mice with an untargeted mass spectrometry-based analysis, Wikoff et al. (2009) showed that a large number of chemical species found in the systemic circulation was unique to conventional mice. In addition, at least 10% of the serum metabolites varied in concentration by at least 50% between the germ-free and conventional mice. This was also nicely illustrated by removing germ-free rats from their aseptic barrier and profiling their urine metabolome using nuclear magnetic resonance spectroscopy. As the rats got conventionalized, their urine was becoming richer in microbiota-derived chemical species, including microbial metabolites and mammalian-microbial co-metabolites, which result from a co-processing from the host and its gut microbiota (Nicholson et al., 2005).

Metabolites from the microbiome are able to signal locally to the various cell populations of the intestinal mucosa, but also to distant organs, including the brain (Sharon et al., 2014; Schroeder and Bäckhed, 2016). Observations that brain and behavior are influenced by the gut microbiota have definitely proven the existence of a gut microbiome-brain axis (Cryan and Dinan, 2012), and evidences that microbiota-derived metabolites may mediate the physiological crosstalk between gut microbiota and brain are increasing. For instance, the short chain fatty acid propionate, produced by microbial degradation of dietary fiber, initiates a gut-brain neural circuit, leading to improved glucose homeostasis in rats (De Vadder et al., 2014). Microbe-related metabolomic signatures may vary between individuals, due to specificities in gut microbiota composition and dietary patterns (Marcobal et al., 2013). In addition, many host and environmental factors, e.g., diseases and medications, can affect gut microbial ecology over a lifetime, leading to alterations in the profile of microbiota-derived metabolites and, hence, to a misconfigured crosstalk between gut microbiota and the host organs, including the brain (Lozupone et al., 2012; Schroeder and Bäckhed, 2016). This concept underlies investigations of microbe-related metabolomic signatures in brain disorders. Urine metabolome profiling with nuclear magnetic resonance spectroscopy showed significant differences between children with autism spectrum disorder and neurotypical controls, specifically perturbations in the patterns of urinary mammalian-microbial co-metabolites, such as dimethylamine, hippurate, and phenylacetylglutamine (Yap et al., 2010). In another study on autism spectrum disorder, children with severe behavioral disabilities had higher urinary levels of 4-methylphenol, a microbiota-derived metabolite produced from tyrosin, than neurotypical controls (Altieri et al., 2011). Interestingly, in studying the relationships between gut microbiota dysbiosis and behavior in a mouse model known to display features of the autism spectrum disorder, Hsiao et al. (2013) identified in these animals a serum metabolite of bacterial origin, the 4-ethylphenylsulfate, which is chemically related to 4-methylphenol, and whose concentration was 46-fold higher than in naive mice. Daily systemic administration of this compound to naive mice for 3 weeks induced in an open-field test an anxiety-like behavior similar to that of the diseased mice. Recently, bacterial degradation of aromatic amino acids was again highlighted, as improved depression scores in patients with irritable bowel syndrome following treatment with the probiotic *Bifidobacterium longum* NCC3001 correlated with decreased urinary levels of 4-methylphenylsulfate (Pinto-Sanchez et al., 2017).

Here, we investigate the effect of another aromatic amino acid derivative, indole, on brain and behavior in rats. Indole is the main metabolite produced by gut bacteria from tryptophan, through the action of the enzyme tryptophanase. While indole is a major intercellular signal within the gut microbial ecosystem (Lee and Lee, 2010), it also interacts with the gut epithelium. Cell culture experiments have shown that it modulates the secretion of glucagon-like peptide 1 by mouse entero-endocrine L cells, and induces genes promoting tight-junction resistance and an anti-inflammatory cytokine profile in the human HCT-8 cell line derived from enterocytes (Bansal et al., 2010; Chimerel et al., 2014). Following absorption, indole is further metabolized by epithelial and hepatic xenobiotic metabolizing enzymes into a family of oxidized and conjugated derivatives (Lee et al., 2015). Among them, oxindole, and isatin are neurodepressant molecules, as indicated by their effects in rats. Indeed, following a systemic injection, both are detected in blood and brain and induce a decrease of the locomotor activity (Bhattacharya et al., 1991; Abel, 1995; Carpenedo et al., 1998; Crumeyrolle-Arias et al., 2003). In addition, depending on the dose, oxindole induces a loss of the righting reflex, a hypotension, and even a reversible coma, while isatin promotes anxiety-like and helplessness behaviors and impairs cognitive functions (Bhattacharya et al., 1991; Abel, 1995; Satyan et al., 1995; Carpenedo et al., 1998 In humans, increased blood levels of oxindole and increased urinary levels of isatin have been reported in hepatic encephalopathy and Parkinson's disease, respectively (Moroni et al., 1998; Hamaue et al., 2000).

Therefore, we hypothesized that an excessive production of indole by the gut microbiota, which may occur due to individual specificities in gut microbiota composition or to a gut microbiota dysbiosis, may result in increased exposure of the brain to oxindole and isatin, and affect behaviors. First, we checked the effect of a systemic injection of oxindole and isatin in rats on the brain levels of these compounds and on the motor activity. Afterwards, we injected indole into the cecum of rats to mimic a microbial acute and high overproduction of this compound; again, we analyzed the brain levels of oxindole and isatin and the motor activity. In addition, as microbiota-derived metabolites, such as butyrate, have been reported to stimulate the vagus nerve (Stilling et al., 2016), we looked for a possible signaling of indole to the brain via this route. Then, we colonized germ-free rats with a wild-type (WT) strain of the indole-producing bacterial species *Escherichia coli*, to mimic a chronic and moderate overproduction of indole. Indeed, as all the bacterial cells in the rat gut belonged to the same species and, thus, were all able to produce indole, we anticipated a higher production of this compound than in conventional rats. The emotional, cognitive and social behaviors of the rats mono-associated with the WT *E. coli* strain were compared to those of germ-free rats colonized with a genetically-engineered counterpart strain unable to produce indole. Finally, to the best of our knowledge, nothing is known about the interindividual variability of the human gut microbiota potential to produce indole. Hence, we analyzed the distribution of the *tna*A gene encoding the tryptophanase enzyme in the latest human microbiota metagenomic catalog (Li et al., 2014), and compared a cohort of 203 individuals with regard to the richness of this gene.

## Materials and methods

### Bacterial strains

The WT *E. coli* BW25113 strain, and the single-gene knock out JW3686 mutant invalidated for the *tnaA* gene described in Baba et al. (2006), were obtained from the National Institute of Genetics (Mishima, Japan). The strains were stored at −80°C in Brain Heart Infusion (BHI) broth added with glycerol (final concentration 40%). For inoculation of germ-free rats, they were grown in BHI broth at 37°C for 4–5 h in a shaking water bath. The cultures were distributed in sterile vials and transferred in sterile conditions into the isolators.

### Animals

The conventional, specific pathogen free, F344 male rats, aged 2–2.5 months, were purchased from Charles River Laboratories France (Saint Germain Nuelles, France). The germ-free F344 rats were obtained locally from the germ-free rodent breeding facility of Anaxem (Germfree animal facilities of the Micalis Institute, France). The conventional rats were housed in a conventional animal room, while the germ-free rats were housed in sterile Plexiglas isolators (Eurobioconcept, Paris, France). The germ-free status was monitored weekly by microscopic examination and aerobic and anaerobic cultures of freshly voided feces. All rats were kept in pairs in Macrolon cages (38 cm long, 22 cm wide, 21 cm high) containing sterile beddings made of wood shavings. They were given a free access to autoclaved tap water and a γ-irradiated (45 kGy) standard diet (R03; Scientific Animal Food and Engineering, Augy, France).The conventional animal room and the isolators were maintained at 20–24°C and on a 12-h light/dark cycle (lights on at 07:30 a.m.).

Experimental procedures with those rats conformed to the European guidelines for the care and use of laboratory animals. They were carried out in accordance with the recommendations of and approved by (approvals #12-068 and #13-002) the ethics committee of the INRA Research Center at Jouy-en-Josas (ethics committee named Comethea, registered by the French Ministry in charge of Research since 2011/06/30 with reference number 45).

### Design of the experiments with the conventional rats

**Figure 1:**
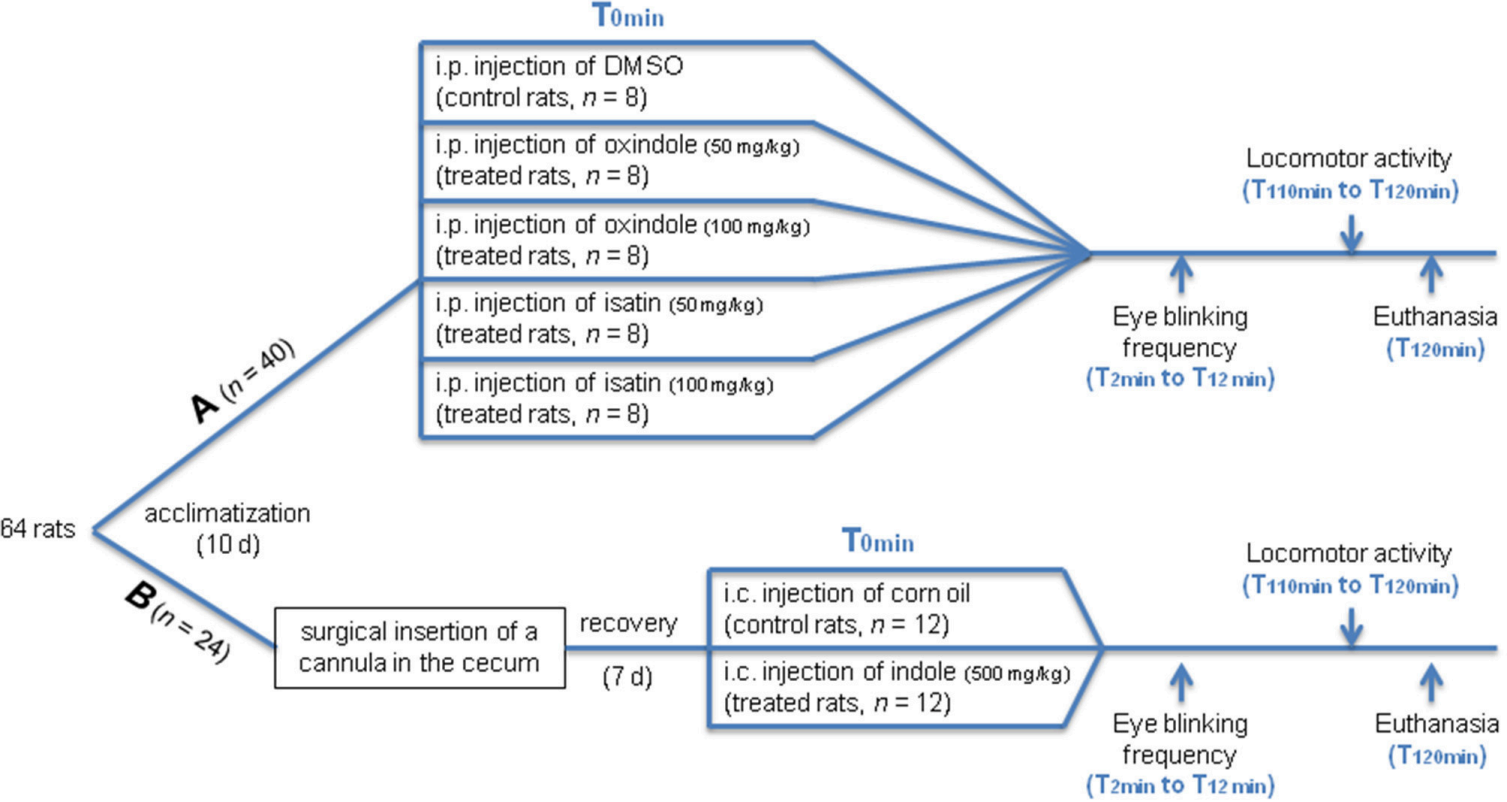
Flow diagram of the experimental design employed in the conventional rats. **(A)** Rats received DMSO (controls) or oxindole or isatin at 2 different doses (treated) through an intra-peritoneal (i.p.) injection. **(B)** Rats received corn oil (controls) or indole (treated) through an intra-cecal (i.c.) injection. Eye blinking frequency and locomotor activity were measured in **(A)** and **(B)** animals.

After a 10-day acclimatization period, the conventional rats received a systemic administration of oxindole or isatin, or an intra-cecal administration of indole. Allocation to either treatment was random. The flow diagram of the experimental design is depicted in Figure 1.

#### Systemic administration of oxindole or isatin

Forty rats received an intraperitoneal (i.p.) injection of 200 μL of oxindole (Sigma-Aldrich, Saint Quentin-Fallavier, France) or isatin (Sigma-Aldrich) dissolved in pure dimethylsulfoxide (DMSO, Sigma-Aldrich) at 50 mg/kg or 100 mg/kg (8 rats/condition), or of 200 μL of pure DMSO alone (*n* = 8; control group). These doses were chosen on the basis of those known to increase the brain oxindole and isatin concentrations and decrease the locomotor activity in rats (Abel, 1995; Carpenedo et al., 1998). To reduce the stress caused by the injection procedure, each rat was handled daily during 1 week prior to the injection. The locomotor activity was measured 110 min after oxindole or isatin injection. For this purpose, the rat was placed in the center of a dimly-lit (10 lux) rectangular open-field arena (90 cm long, 70 cm wide, 60 cm high) with a squared floor; the number of squares crossed, reflecting the traveled distance, and the number of rearings and defecations were recorded for 10 min. In addition, as preliminary experiments showed that eye blinking frequency was enhanced following intra-cecal administration of indole, the number of eye blinks was also measured in oxindole and isatin-treated rats, for 10 min, starting 2 min after the i.p. injection. The rats were killed by decapitation immediately after the locomotor activity test.

#### Intra-cecal administration of indole

Twenty-four rats were anesthetized with isoflurane (Aerrane®, Baxter, Guyancourt, France) and given an analgesic (flunixine, 2 mg/kg, subcutaneous injection, Finadyne®, Schering-Plough, Levallois-Perret, France). A small incision was made in the abdominal and cecal walls and a flexible Tygon® cannula (inner diameter 3.4 mm; outer diameter 5.4 mm) was introduced into the cecal lumen and secured to the cecal wall with polypropylene suture (Prolene™, Ethicon, Issy les Moulineaux, France).The abdominal muscle was stitched with polypropylene thread (Prolene™, Ethicon). Then the length of cannula tubing protruding from this entry point was subcutaneously worked/brought around from the incision point in the abdominal area to the back of the animal, to a point between the shoulders, where a 2-cm length was then led outside the skin. Once the cannula was thus positioned, the skin incisions in the abdominal and shoulder areas were stitched shut with silk thread (Ethicon). The open end of the cannula was then heat sealed. The length of the cannula protruding outside the skin from between the shoulders was wrapped with thin-gauge brass wire to prevent the rat from gnawing the cannula. The rats were carefully watched over until the anesthetic had worn off. Following this, the analgesic flunixine was injected once a day for a couple of days after surgery. The general state of health, the water and food consumption, and the proper healing of the sutured areas were monitored every day for a week. The quantity and appearance of the fecal pellets were taken as biomarkers of a normal intestinal transit and checked for the same time.

Thereafter, the rats were injected into the cecum via the cannula, either with 1 mL of indole (Sigma-Aldrich) dissolved in corn oil (Sigma-Aldrich) at 500 mg/kg (*n* = 12), or with corn oil alone (*n* = 12; control group). The dose we used was extrapolated from Carpenedo et al. (1998) to target brain and obtain behavior modifications. The number of eye blinks and the locomotor activity were each measured for 10 min, starting 2 and 110 min after the indole administration, respectively. The rats were killed by decapitation immediately after the locomotor activity test.

### Design of the experiment with the gnotobiotic rats

**Figure 2:**
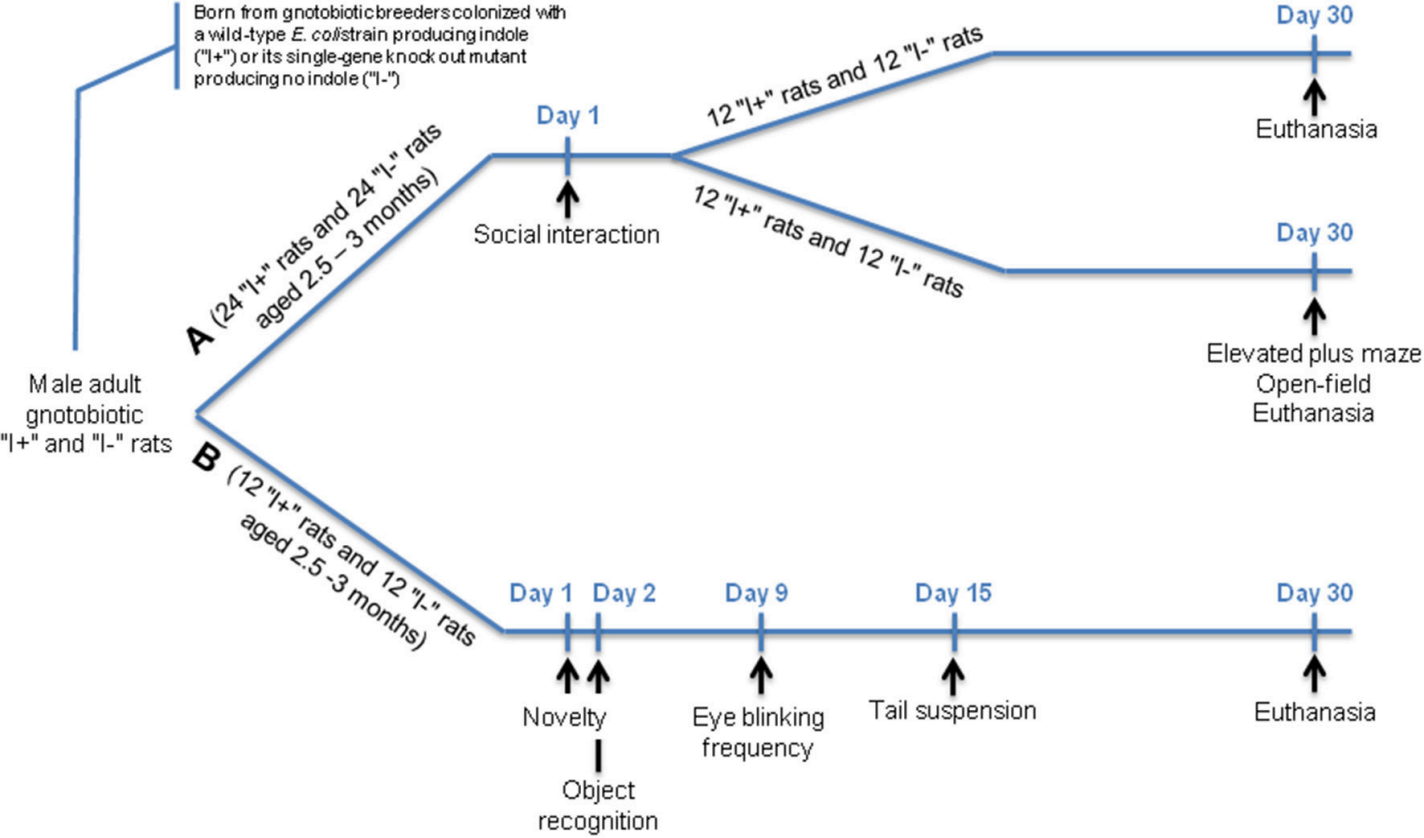
Flow diagram of the experimental design employed in the gnotobiotic rats**. (A)** A first series of rats was subjected to an investigation of social behavior (social interaction test) and anxiety-like behavior (elevated plus maze and open-field tests). **(B)** As differences occurred in the anxiety-like behavior of the **(A)** rats, further behavioral analyses were carried out in a second series of rats: confirmation of the anxiety-like behavior through a novelty test, exploration of memory (object recognition task) and of despair behavior (tail suspension test). The eye blinking frequency was also measured in these rats. All rats were aged 2.5–3 months when the behavioral tests started (Day 1).

Germ-free adult male and female F344 rats were inoculated intragastrically with 1 mL of a bacterial culture of the BW25113 or JW3686 strains. These animals were mated 10 days later. This procedure allowed the offspring to be colonized from birth onwards with the bacterial strains of interest. The bacterial status in the breeders and in the offspring was checked weekly by microscopic examination, and cultures, of freshly voided feces. Among the offspring, two experimental series with adult male rats were made, and with two types of rats: one type was mono-associated with the WT BW25113 strain and named “I+” rats, and the other type was mono-associated with the mutant JW3686 strain and named “I−” rats, as depicted in Figure 2. In the first experimental series, one group of 24 type “I+” rats and one group of 24 type “I−” rats were subjected to a social interaction test at 2.5–3 months of age. One month later, two tests aimed at exploring anxiety-like behavior—namely an elevated plus maze and an open-field tests—were carried out in half of each “I+” and “I−” group. These rats were killed by decapitation immediately after the open-field challenge, together with those that had not undergone the anxiety tests and had served thus as non-stressed controls for blood corticosterone measurement. As differences occurred in the anxiety-like behavior of “I+” and “I−”rats, further behavioral tests were carried out in a second series of 12 “I+” and 12 “I−” rats: at 2.5–3 months of age, a novelty test and an object recognition task, aimed at exploring anxiety-like behavior and memory, respectively; then at 3–3.5 months of age, a tail suspension test aimed at exploring despair behavior. The rats were killed by decapitation a couple of weeks later, at the age of 3.5–4 months. In addition, as an increase in eye blinking frequency had been observed in the conventional rats treated with indole, the number of spontaneous eye blinks was also measured in the gnotobiotic rats during 5 min, 1 week after the object recognition task.

### Behavioral tests in the gnotobiotic rats

The behavioral tests were carried out at time points indicated in Figure 2. Within a day, they were performed between 10:00 a.m. and 4:00 p.m. The tests were videotaped, and videos were then analyzed blindly by two independent observers, whose results were averaged. All tests were carried out inside the isolators, except for the elevated plus maze and the open-field tests, whose devices were too large to be placed in an isolator. Therefore, these tests were carried out as follows: rats were removed from the isolators and housed in a conventional room for 2 h to allow them to adapt prior to the tests (Crumeyrolle-Arias et al., 2014).

#### Social interactions

This test consisted in bringing a rat to an unknown sex-, age-, and weight-matched partner for 10 min, and in measuring the time spent in social contact (sniffing, following, crawling under and crawling over) for the pair, and in self-grooming for each rat. We adapted this test from File and Hyde (1978) as described in Crumeyrolle-Arias et al. (2014).

#### Elevated plus maze

The test apparatus, initially described by Handley and Mithani (1984), was a dark gray maze in the shape of a cross composed of 4 arms (50 cm long, 10 cm wide). Two opposite arms were surrounded on 3 faces by 50 cm high walls (closed arms), while the other two opposite arms were open (open arms). The maze was raised to a height of 70 cm from the floor and was illuminated from the top. To encourage the rat to explore the open arms, which are considered as an aversive area, these arms were less lit than the closed ones (50 lux vs. 150 lux). At the beginning of the test, the rat was placed in the intersection square, facing an open arm, then the following behaviors were recorded for 5 min: number of entries into the open and closed arms (an entry was counted when the rat placed all four paws into an arm), number of visits at the end of the open arms, number of head dippings, time spent in the open and closed arms, hesitation time (i.e., time spent in the intersection square), immobility time, number of rearings, stretchings, and self-groomings.

#### Open-field

We adapted this test from (Crawley, 1985)as described previously in Crumeyrolle-Arias et al. (2014). It was carried out just after the elevated plus maze test. Briefly, the rat was placed in a corner of a rectangular open-field arena (90 cm long, 70 cm wide, 60 cm high), with a squared floor and strongly lit in the center (500 lux). The following parameters were recorded for 6 min: time to move from the initial corner (latency time), number of squares crossed (traveled distance), number of visits to the aversive central area, number of rearings, self-groomings and defecations.

#### Novelty

This test was performed during the habituation period preceding the object recognition task. The rat was placed in a dimly lit dark gray open-box (79 cm long, 49 cm wide, 30 cm high) at a distance of an unknown object. The time to go near to the object to explore it (latency time), the time spent exploring the object (i.e., nose directed toward the object at a distance less than 2 cm), and the locomotor activity were monitored for 10 min (Ennaceur et al., 2005).

#### Object recognition task

We subjected the rats to this task the day after the novelty test, which had allowed them to get used to the test arena. The rat was placed in the arena in the presence of two identical objects (A1 and A2) and left free to explore these objects for 3 min (sample phase). After a 1-h rest period in its home cage, the rat was brought back for 3 min in the arena (test phase), where one of the two objects was identical to the previous ones (A3) and the second was new (B1). To avoid possible bias due to the position of the objects, the B1 object was placed to the right of the A3 object for half of the rats, and *vice versa* for the other half. The time of exploration of each object (i.e., nose directed toward the object at a distance less than 2 cm) was measured, and an exploration index, defined as the ratio of the time spent exploring the new object to the total time spent exploring the two objects, was calculated (Ennaceur and Delacour, 1988).

#### Tail suspension

The rat was suspended by the tail with an adhesive tape (Elastoplast®) to a stainless steel girder positioned 50 cm above the ground of the isolator. The suspension lasted 6 min, during which the rat showed periods of movements and immobility. The duration of the immobility, defined as the absence of movement except for respiration and whiskers' movements, was measured and taken as an index of despair behavior (Chermat et al., 1986).

### Euthanasia and fluid and tissue collection

The rats were killed by decapitation. The trunkal blood was collected in a plastic tube coated with an anticoagulant solution (sodium EDTA 0.5 M). After centrifugation (2,500 g, 20 min, 4°C), the plasma was divided into CryoTubes® and frozen at −80°C until oxindole and isatin analysis (conventional rats), or corticosterone assays (gnotobiotic rats). The brain was removed from the cranium, placed 1 min at −30°C in isopentane, then stored at −80°C until oxindole and isatin analysis (conventional and gnotobiotic rats) or c-Fos protein assay (conventional rats). Whenever possible, the urine remaining in the bladder was collected and stored in CryoTubes® at −80°C for indoxylsulfate analysis. In the gnotobiotic rats, the cecal content was collected and frozen at −80°C to measure tryptophan and indole concentrations.

### Corticosterone analysis in plasma

Corticosterone was measured in duplicate by radioimmunoassay, using a corticosterone double antibody ^125^I RIA kit for rats and mice (MP Biomedicals, Illkirch-Graffenstaden, France). Briefly, diluted plasma samples were incubated for 2 h at room temperature with ^125^I radiolabeled corticosterone and with anti-corticosterone antibodies generated in rabbits. At the end of incubation, addition of a mixture of polyethylene glycol and goat anti-rabbit gamma globulins precipitated the antibody bound antigens, and the amount of radioactivity in the precipitate was counted with a gamma counter. This amount was inversely correlated with the quantity of corticosterone present in the sample. Quantification was done using standard curves constructed with calibrators containing known amounts of corticosterone.

### Analysis of tryptophan, indole, and its metabolites

Tryptophan and indole in cecal contents, indoxylsulfate in urine and oxindole and isatin in plasma and brain were analyzed by HPLC using a 2690 autosampler and separation module equipped with a 474 fluorescence detector and a 996 photodiode array detector (Waters, Saint-Quentin-en-Yvelines, France). Separation of tryptophan, indole, oxindole and isatin was carried out in a reversed-phase column packed with LiChrospher® 100RP-18e (5 μm, 250 × 4.3 mm; VWR, Strasbourg, France), equipped with a guard column LiChrospher® 100 RP-18e (5 μm, 4 × 4 mm; VWR). Separation of indoxylsulfate was carried out in a Kinetex® reversed-phase C18 column (5 μm, 250 × 4.6 mm; Phenomenex, Le Pecq, France), equipped with a security guard ULTRA cartridge UHPLC C18 (Phenomenex). We used fluorescence detection for tryptophan, indole and indoxylsulfate (Krstulovic and Matzura, 1979; Deguchi et al., 2003), and diode-array detection for oxindole and isatin in plasma (Manabe et al., 1997) and brain (Carpenedo et al., 1998; Igosheva et al., 2004). The HPLC system was run and the calculations were performed with the Millenium® software (Waters).

#### Tryptophan and indole analysis in cecal contents

Frozen cecal contents of the gnotobiotic rats were thawed, weighed and 10-fold diluted (w/v) in 50 mM PBS pH 7.0. After homogenization, the cecal suspension was centrifuged (8,000 g, 10 min, 4°C) and the supernatant was collected. The pellet was resuspended and centrifuged again twice, and the 3 supernatants were pooled for injection onto the HPLC column (sample volume: 50 μL). Elution was isocratic (55% methanol and 45% ultrapure water) at a flow rate of 0.5 mL/min. The excitation and emission wavelengths of the detector were set at 285 and 320 nm, respectively. Tryptophan and indole eluted at 5.3 and 16.7 min, respectively, and were quantified using external standard curves. Results were expressed as nmol/g dry matter (DM). DM was determined by weighing cecal content samples before and after 24-h freeze-drying (VirTis Lyophilizer, SP Scientific, Gardiner, NY, USA).

#### Indoxylsulfate analysis in urine

Urine samples were thawed and clarified by centrifugation (9,000 g, 15 min, 4°C). The supernatants were 10- to 100-fold diluted in acetate buffer (50 mM, pH 4.0) and 100-μL samples were injected onto the HPLC column. Elution was isocratic (25% acetonitrile and 75% 50 mM acetate buffer pH 4.0) at a flow rate of 1 mL/min. The excitation and emission wavelengths of the detector were set at 280 and 375 nm, respectively. Indoxylsulfate eluted at 6.9 min and was quantified using an external standard curve.

#### Oxindole and isatin analysis in plasma

Five hundred microliter of plasma were mixed with 1 mL of 0.1 N hydrochloric acid, heated at 99°C for 10 min, then cooled under tap water. A liquid-liquid extraction was carried out by adding 3 mL of ethyl acetate, shaking vigorously and spinning the mixture. The organic phase was collected and evaporated under N_2_. The residue was suspended in 100 μL methanol and 100 μL 0.1 N hydrochloric acid. After centrifugation (8,000 g, 10 min, room temperature), the supernatant was collected and injected onto the HPLC column (sample volume: 100 μL). Elution was isocratic (55% methanol and 45% ultrapure water) at a flow rate of 0.5 mL/min, and was monitored at 244 nm. Isatin and oxindole eluted at 6.6 and 8.1 min, respectively, and were quantified using external standard curves.

#### Oxindole and isatin analysis in brain

Brains were thawed on ice and cut into two hemispheres. Oxindole and isatin were analyzed separately in one hemisphere, alternately the right one and the left one within a same group of rats to avoid a possible lateralization bias. For oxindole, the half-brain was homogenized at 4°C with a Potter-Elvehjem grinder in 2 volumes of 0.4 N perchloric acid and centrifuged (15,000 g, 20 min, 4°C). The supernatant was extracted with chloroform. After shaking and spinning, the organic phase was evaporated under N_2_ and the residue was suspended in 200 μL of 0.4 N perchloric acid. The mixture was centrifuged (8,000 g, 10 min, room temperature) and the supernatant was collected for HPLC analysis. For isatin, the half-brain was homogenized at 4°C with a Potter-Elvehjem grinder in 2 volumes of 0.1 N hydrochloric acid added with 5-methyl-isatin (internal standard; Sigma-Aldrich) and centrifuged (3,000 g, 20 min, 4°C). The supernatant was extracted twice with ethyl acetate and the two organic phases were pooled and evaporated under N_2_. The residue was suspended in 100 μL of methanol and 100 μL of 0.1 N hydrochloric acid; the mixture was centrifuged (8,000 g, 10 min, room temperature) and the supernatant was collected for HPLC analysis. For oxindole, 100-μL samples were separated in isocratic conditions (85% of a 50 mM acetate buffer pH 4.4 and 15% of acetonitrile) at a flow rate of 1 mL/min. The elution was monitored at 244 nm, the retention time was 12.6 min, and the quantification was done using an external standard curve. For isatin, the injection volume was 75 μL, and the separation was performed by gradient elution with 50 mM phosphate buffer pH 7.4 as solvent A and acetonitrile as solvent B at a flow rate of 1 mL/min. The gradient program was as follows: 15% B, 0–10 min; 15% −35% B, 10–20 min; 35–15% B, 20–21 min. The elution was monitored at 244 nm, the retention times were 10.7 and 19.6 min for isatin and 5-methyl-isatin, respectively, and isatin quantification was done with the internal standard.

### c-Fos protein immunohistochemistry in the hindbrain dorsal vagal complex (DVC)

Serial 20-μm-thick coronal brain sections were cut at -20°C with a cryostat microtome (Leica CM3050S, Leica Microsystems, Heerbrugg, Switzerland). Those covering the DVC area in the hindbrain, namely from Bregma−12.50 mm to −14.60 mm according to Paxinos and Watson (2005), were mounted onto Superfrost Plus microscope slides (Menzel-Gläser, Braunschweig, Germany) and stored at −80°C. c-Fos protein immunohistochemistry was adapted from the methods of Sundquist and Nisenbaum (2005) and Mingam et al. (2008). Briefly, the frozen sections were thawed, fixed in 4% paraformaldehyde in PBS for 15 min at room temperature, and washed thrice in PBS. Sections were then covered for 30 min with 0.3% H_2_O_2_ to saturate the tissue peroxidases, then rinsed thrice with PBS. In order to permeabilize the tissue and to reduce the non-specific reactions, the sections were covered with 500 μL of PBS containing 0.25% Triton and 3% normal donkey serum for 2 h at room temperature. Then, the sections were incubated overnight at 4°C with 400 μL of goat anti-c-Fos antibody solution (Santa Cruz Biotechnology, Clinisciences, Nanterre, France) 500-fold diluted in PBS containing 0.25% Triton and 3% normal donkey serum. After 3 washes in PBS (10 min/bath), sections were incubated for 1 h at room temperature with 400 μL of biotinylated anti-goat IgG solution (Vector Laboratories, Clinisciences) 200-fold diluted in PBS containing 0.25% Triton and 3% normal donkey serum. The sections were soaked thrice in PBS (10 min/bath), then incubated for 1 h at room temperature with 600 μL of ABC reagent kit “Vectastain® Elite ABC” (Clinisciences). The sections were again soaked thrice in PBS (10 min/bath), and covered for 20 min at room temperature with 600 μL of a solution of diaminobenzidine (0.02 mg/mL) containing 0.035% H_2_O_2_. The reaction was stopped by soaking the slides twice in PBS (5 min/bath). The slides were then air-dried and dehydrated by consecutive 3-min exposures to ethanol bathes (70°: once, 95°: once, 100°: twice) and Ottix bathes (twice; Microm Microtech, Brignais, France). Finally, the slides were covered with Diamount mounting medium (Microm Microtech), and observed through a microscope at magnification X63 (Leica DM R, Leica Microsystems). For each rat, the number of labeled cells was counted on two sections, at Bregma −13.38 and −13.68 mm, blindly of the rat group.

### Analysis of the tryptophanases present in the human intestinal microbiota

In order to identify the tryptophanase-like proteins putatively encoded by the bacterial genes present in the human intestinal microbiota, we used the data from the latest human microbiota metagenomic catalog (Li et al., 2014). At first, we extracted all the prokaryotic TnaA-like proteins existing in the public databases (Swissprot, Pfam and NCBI). This list was manually edited in order to check the BLAST homology between the sequences retrieved from databases and the TnaA proteins well characterized in the literature (Yoshida et al., 2009; Sasaki-Imamura et al., 2011); all retrieved proteins displaying at least 50% of homology on their full length with the reference TnaA were kept in the list. The redundancy was removed using an internal developed script based on the identity of the TnaA sequences. The curated TnaA sequences list was then used to extract the homologous sequences contained in the human metagenomic catalog. A multiple sequence alignment was then performed using Clustal W (Thompson et al., 1994), an overall phylogenetic tree was inferred using MAFFT scripts (Katoh et al., 2002).

### Statistical analyses

Most of the data did not show a normal distribution. Therefore, to be consistent, we analyzed all the data with non-parametric tests, and expressed all of them as median(interquartile range). The level of significance was set at *P* < 0.05. Calculations were performed with the GraphPad Prism software (version 5.04, La Jolla, CA, USA).

## Results

### Systemic administration of oxindole or isatin to conventional rats increased the brain concentrations of these molecules and decreased the motor activity

**Figure 3:**
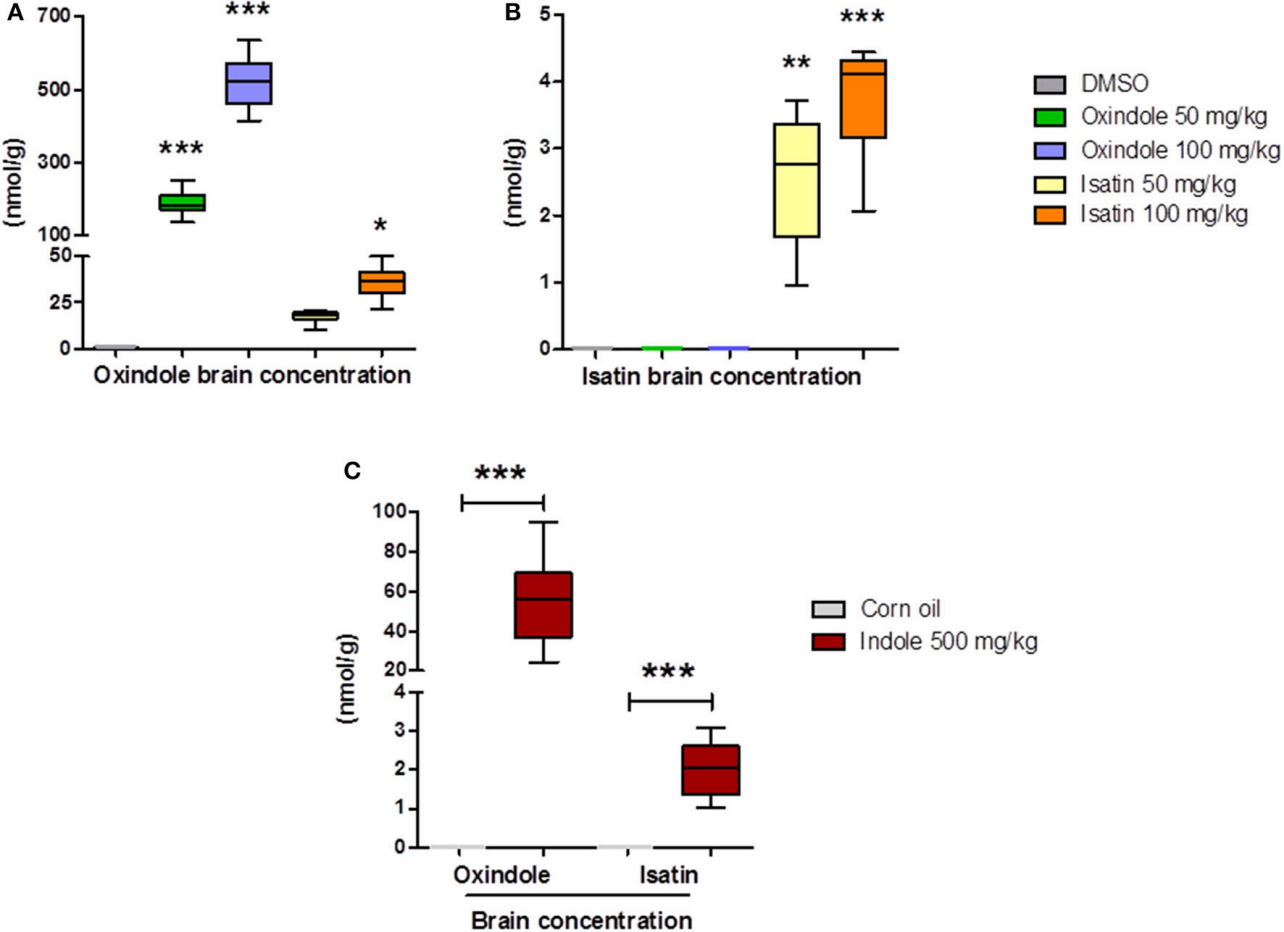
Oxindole and isatin concentrations in the brain of conventional rats treated with oxindole, isatin or indole**. (A)** Oxindole and **(B)** Isatin brain concentrations (nmol/g) after i.p. injection of oxindole or isatin (50 and 100 mg/kg) or of the DMSO vehicle (*n* = 7–8 rats/group, Kruskal-Wallis test followed by a Dunn's multiple comparison test). **(C)** Oxindole and isatin brain concentrations (nmol/g) after intra-cecal administration of indole (500 mg/kg) or of the corn oil vehicle (*n* = 11 rats/group, Mann-Whitney test). Data are medians with interquartile ranges. ^*^P < 0.05, ^**^P < 0.01, ^***^P < 0.001, compared with the control group.

As expected, neither oxindole nor isatin could be detected in the brain of the control rats which had received an i.p. injection of the DMSO vehicle. Compared with the control rats, oxindole administration at 50 or 100 mg/kg (i.e., 375 or 750 μmol/kg) led to high brain concentrations of this molecule, in the range of 100–600 nmol/g tissue according to the dose, 2 h after the i.p. injection (Figure 3). Similarly, isatin administration at both concentrations (i.e., 340 or 680 μmol/kg) led to the presence of isatin in the brain (Figure 3). However, the order of magnitude of isatin brain concentrations in isatin-treated rats was 100-fold less than oxindole concentrations in oxindole-treated rats. In addition, substantial amounts of oxindole were measured in the brain of isatin-treated rats, suggesting a metabolic conversion of isatin to oxindole.

**Figure 4:**
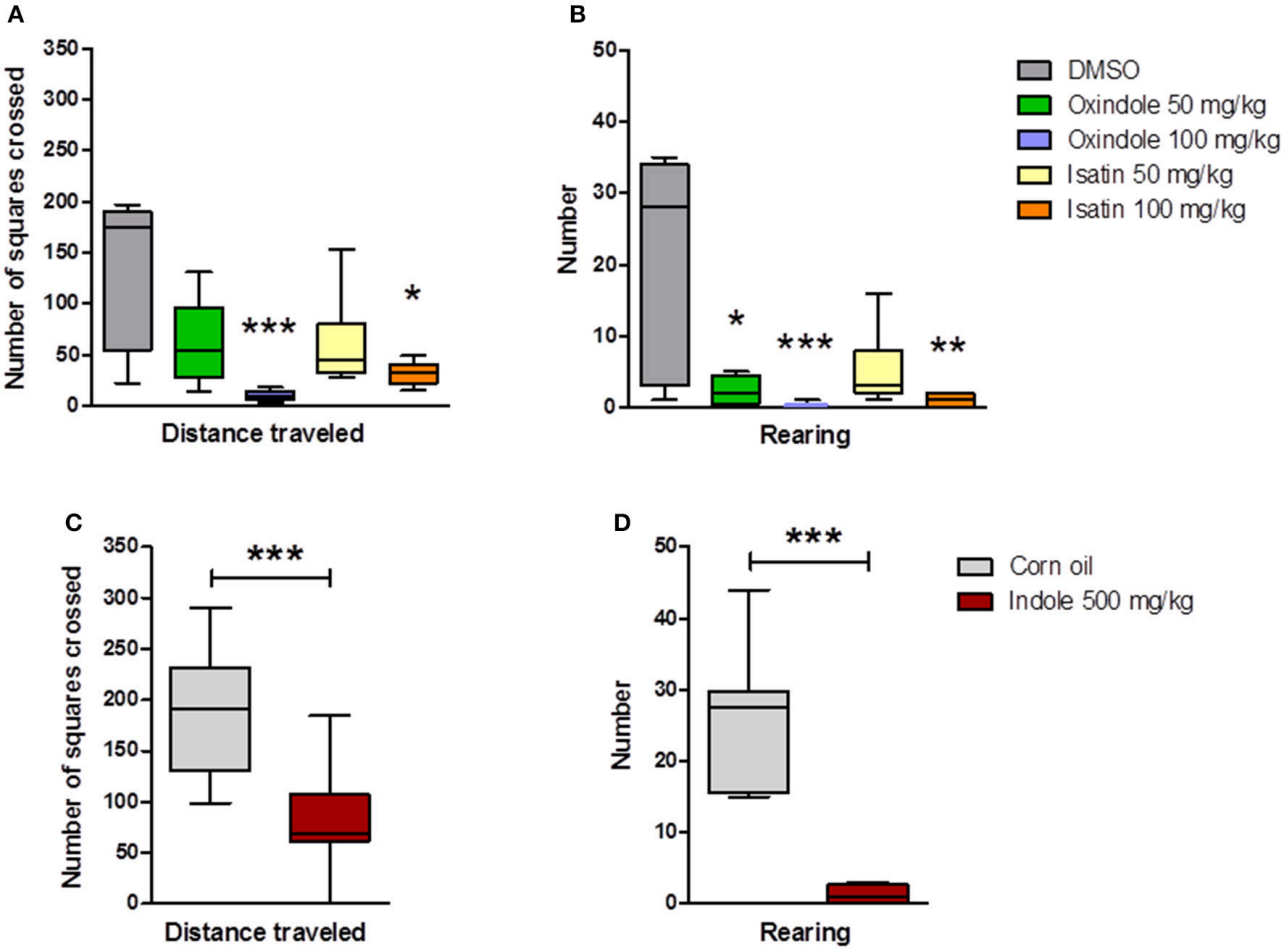
Motor activity (locomotion and rearings) measured in an open-field in conventional rats treated with oxindole, isatin or indole. **(A)** Distance traveled and **(B)** Number of rearings after i.p. injection of oxindole or isatin (50 and 100 mg/kg) or of the DMSO vehicle (*n* = 7–8 rats/group, Kruskal-Wallis test followed by a Dunn's multiple comparison test). **(C)** Distance traveled and number of rearings after intra-cecal administration of indole (500 mg/kg) or of the corn oil vehicle (*n* = 12 rats/group, Mann-Whitney test). Data are medians with interquartile ranges. ^*^P < 0.05, ^**^P < 0.01, ^***^P < 0.001, compared with the control group.

**Figure 5:**
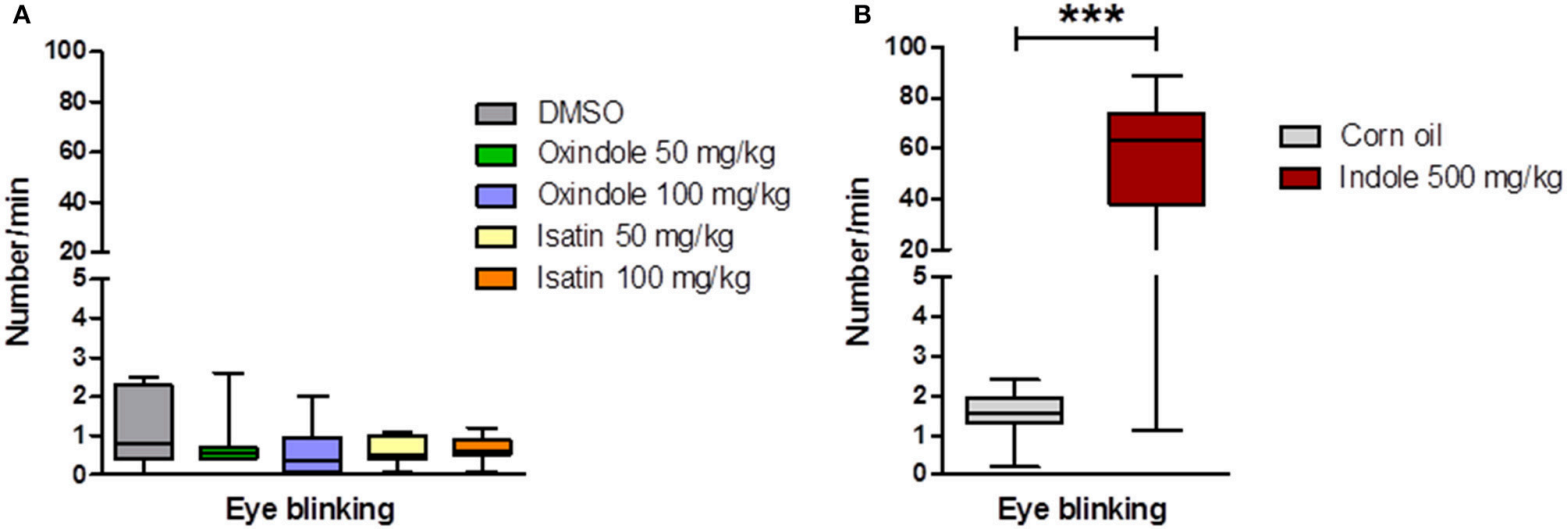
Eye blinking frequency in conventional rats treated with oxindole, isatin, or indole**. (A)** Number of eye blinkings/min during 10 min, from 2 min after i.p. injection of oxindole or isatin (50 and 100 mg/kg) or of the DMSO vehicle (*n* = 7–8 rats/group, Kruskal-Wallis test followed by a Dunn's multiple comparison test). **(B)** Number of eye blinkings/min during 10 min, from 2 min after intra-cecal injection of indole (500 mg/kg) or of the corn oil vehicle (*n* = 12 rats/group, Mann-Whitney test). Data are medians with interquartile ranges. ^***^P < 0.001.

Oxindole and isatin administration influenced the motor activity, as assessed for 10 min in a dimly-lit open arena, 2 h after the i.p. injection. The 100 mg/kg dose of either molecule led to a dramatic decrease of the traveled distance and of the number of rearings (Figure 4). At 50 mg/kg, only the number of rearings in oxindole-treated rats was significantly reduced (Figure 4). In contrast, compared with the control rats, neither oxindole nor isatin modified the eye blinking frequency, regardless of the dose (Figure 5).

### Intra-cecal administration of indole to conventional rats increased oxindole and isatin in the brain, activated the vagus nerve and decreased the motor activity

To ascertain that indole injected in the cecum (500 mg/kg) was actually absorbed by the intestinal mucosa and further metabolized by the liver xenobiotic metabolizing enzymes, we sampled urine in the bladder of the rats at the time of killing, to measure indoxylsulfate, the main indole metabolite excreted by this route. Urine collection was possible for 8 out of the 12 indole-treated rats, and for 6 out of the 12 control rats. The bladder of the other rats was empty. The urinary concentration of indoxylsulfate was much higher (Mann-Whitney test; *P* < 0.001) in the indole-treated rats than in the control rats, i.e., 10.9(12.9) vs. 0.5(0.8) μmol/mL [median(interquartile range)].

Then, oxindole and isatin were measured in plasma and brain. As expected, these molecules were not detected in the plasma of the control rats, while the concentrations of oxindole and isatin in the plasma of the treated rats were 17(5) and 2(1) nmol/mL, respectively. In the brain, traces of oxindole were detected in 3 control rats, and traces of isatin in another one. By contrast, the brain of the treated rats contained around 55 nmol/g oxindole and around 2 nmol/g isatin (Figure 3).

**Figure 6:**
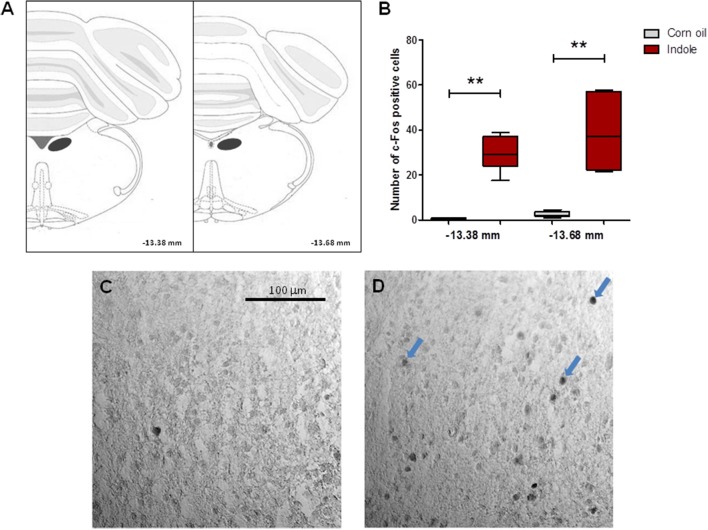
c-Fos immunohistochemistry in the brain of conventional rats treated with indole. **(A)** c-Fos immunolabeled cells were counted in the DVC (area indicated by a dark oval) at two levels, −13.38 and −13.68 mm from the bregma. **(B)** Number of c-Fos positive cells in the counting areas after intra-cecal administration of indole (500 mg/kg) or of the corn oil vehicle (*n* = 5–6 rats/group, Mann-Whitney test). Data are medians with interquartile ranges. ^**^P < 0.01. **(C,D)** Photomicrographs illustrating c-Fos immunoreactivity in the DVC of the corn oil and indole treated rats, respectively. Arrows indicate c-Fos immunolabeled cells.

Investigation of the motor activity showed that the traveled distance and the number of rearings were considerably decreased in the indole-treated rats, compared with the control counterparts (Figure 4). Interestingly, this decrease was in the same order of magnitude as those observed after systemic administration of oxindole or isatin. Intra-cecal injection of indole also led to a dramatic increase of the eye blinking frequency (Figure 5), and of the number of cells expressing the c-Fos protein - taken as a biomarker of neuronal activation - in the hindbrain DVC (Figure 6).

### Validation of a rat model mono-associated with a bacterial strain expressing or deficient for the tryptophanase activity

To go further, we selected a WT *E. coli* strain expressing the tryptophanase (TnaA) activity able to produce indole from tryptophan (I+) and its derived mutated strain inactivated in the *tnaA* gene and consequently unable to produce indole from tryptophan (I–). We ascertained that both bacterial strains colonized the gut of the gnotobiotic rats at the same level. This was indeed the case as the cecal concentrations of the WT in the “I+” rats, and of the mutant in the “I−” rats, were 1.2(1.3).10^9^ and 2.3(1.3).10^9^ CFU/g fresh cecal content, respectively (Mann-Whitney test; *P* > 0.05).

We checked that the rats mono-associated with the WT produced indole, while those mono-associated with the mutant did not, by measuring the tryptophan and indole concentrations in the cecal contents. As expected, the cecal concentration of indole was null in the “I−” rats, while it reached 123(57) nmol/g DM of cecal content in the “I+” rats. On the opposite, tryptophan concentration was 3.6(1.0) μmol/g DM in the cecum of the “I−” rats, whereas no tryptophan could be detected in the cecal content of the “I+” rats. The indole cecal concentration in the “I+” rats reflected an overproduction of indole, as measurements in conventional SPF male F344 rats of the same age and fed on the same diet indicated a 4-fold lower concentration in the latter ones (data not shown).

The indole produced in the hindgut by the WT resulted in the circulation of mammalian-microbial indole derivatives in the body, as shown by the presence of indoxylsulfate in the urine of the “I+” rats. Urine could be collected at the time of killing in 12 “I+” rats and in 9 “I−” rats. Indoxylsulfate concentration was 800(915) nmol/mL in the “I+” rats while it was consistently null in the “I−” rats.

However, the microbial production of indole in the hindgut was insufficient to lead to substantial amounts of oxindole and isatin in the brain. Indeed, we could not detect isatin in the brain of the “I+” rats, and we observed only traces of oxindole in three of them. As expected, there was no oxindole or isatin in the brain of the “I−” rats.

### The “I+” rats displayed greater social contacts than the “I−” rats

**Figure 7:**
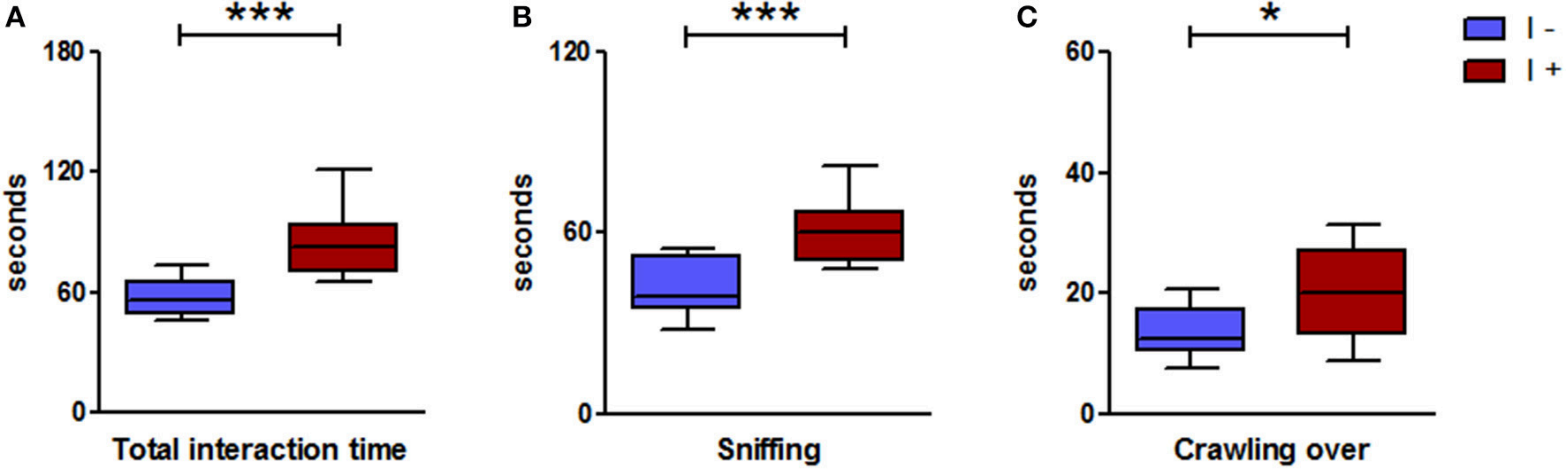
Social interactions in gnotobiotic rats mono-associated with the WT *E. coli* strain (“I+” rats) or with the mutant inactivated for the *tnaA* gene (“I−” rats). **(A)** Duration of the total interaction behavior of pairs of “I−” and “I+” rats, **(B,C)** Duration of the two main behaviors, respectively sniffing and crawling over (*n* = 12 pairs/group, Mann-Whitney test). Data are medians with interquartile ranges. ^*^P < 0.05, ^***^P < 0.001.

Overall, the “I+” rats spent more time in social interactions than their “I−” counterparts (Figure 7). Specifically, they spent more time in sniffing and crawling over behaviors (Figure 7), while no significant difference occurred between the two groups with regard to following [2.10(3.30) s vs. 0.95(1.90) s] and crawling under [1.35(0.17) s vs. 0.40(1.15) s] behaviors (Mann-Whitney test; *n* = 12 pairs of rats; *P* > 0.05). No significant difference was observed for the self-grooming behavior: 115.8(112.6) s in the “I+” rats and 133.8(105.3) s in the “I−” rats (Mann-Whitney test; *n* = 24; *P* > 0.05).

### The “I+” rats had greater anxiety-like and despair behaviors than the “I−” rats

#### Elevated plus-maze test

**Figure 8:**
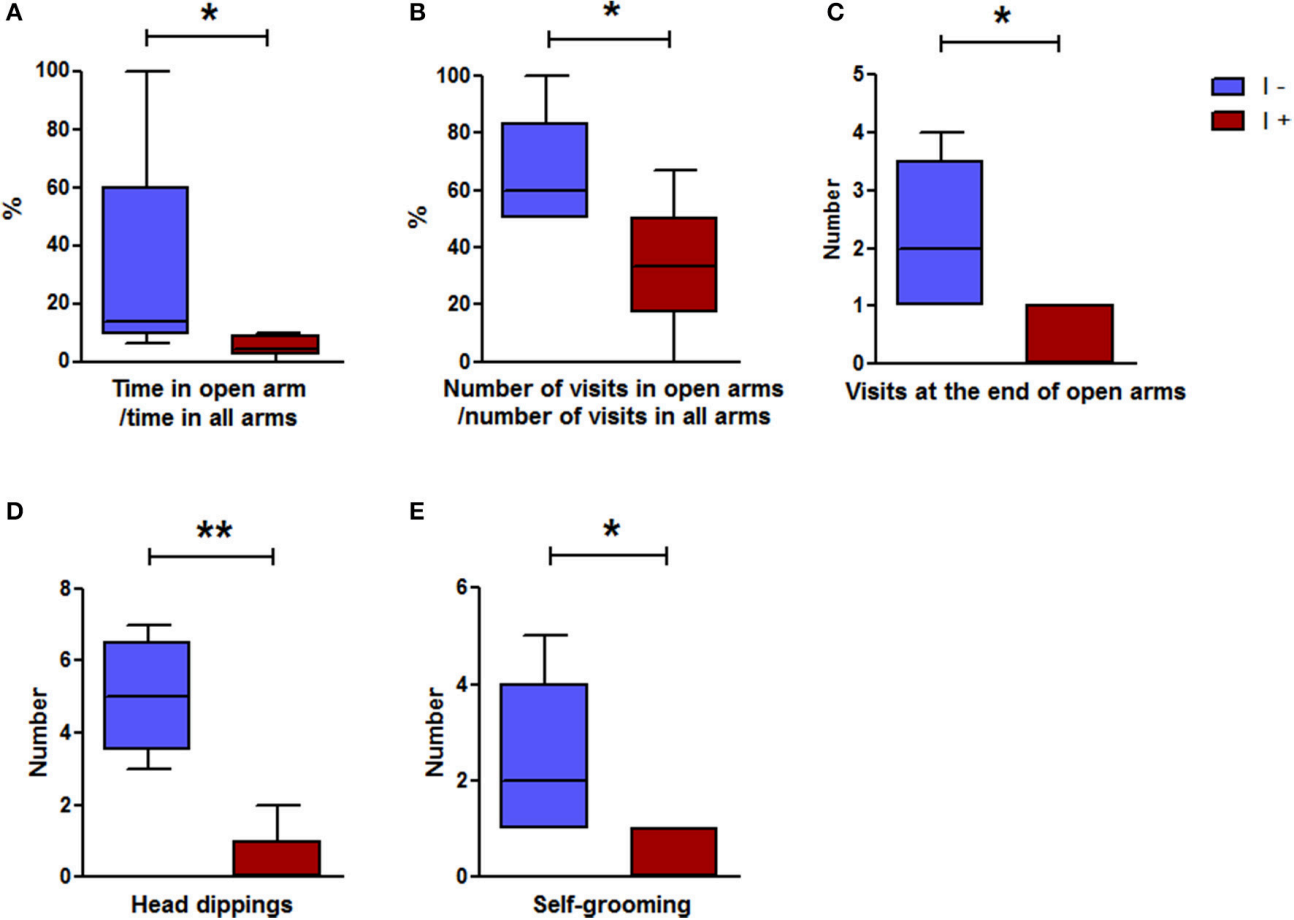
Elevated plus maze test in gnotobiotic rats mono-associated with the WT *E. coli* strain (“I+” rats) or with the mutant inactivated for the *tnaA* gene (“I−” rats). **(A)** Ratio of the time spent in open arms divided by the time spent in all arms, **(B)** Ratio of the number of visits in open arms divided by the number of visits in all arms, **(C)** Number of visits at the end of open arms, **(D)** Number of head dippings, **(E)** Number of self-groomings (*n* = 5–7 rats/group, Mann-Whitney test). Data are medians with interquartile ranges. ^*^P < 0.05, ^**^P < 0.01.

Usually, it is considered that a rat has successfully performed this test if the number of visits in at least one arm is ≥3. Based on this criterion, 7 out of the 12 “I+” rats and 5 out of the 12 “I−” rats were retained for behavior comparison, which showed that the “I+” rats had a greater anxiety-like behavior than the “I−” rats. They spent significantly less time in the open arms, they made fewer entries in the open arms, and paid fewer visits to the end part of these arms; they also made fewer head dippings and self-groomings (Figure 8). No significant difference occurred between the two groups with regard to (i) the number of rearings [3(3) in the “I+” rats and 4(8) in the “I−” rats], (ii) the number of stretchings [4(4) in the “I+” rats and 1(1) in the “I−” rats], (iii) the hesitation time [78.2(86.7) s in the “I+” rats and 79.8(152.2) s in the “I−” rats], and (iv) the immobility time [136.6(132.7) s in the “I+” rats and 56.5(119.3) s in the “I−” rats].

#### Open-field test

**Figure 9:**
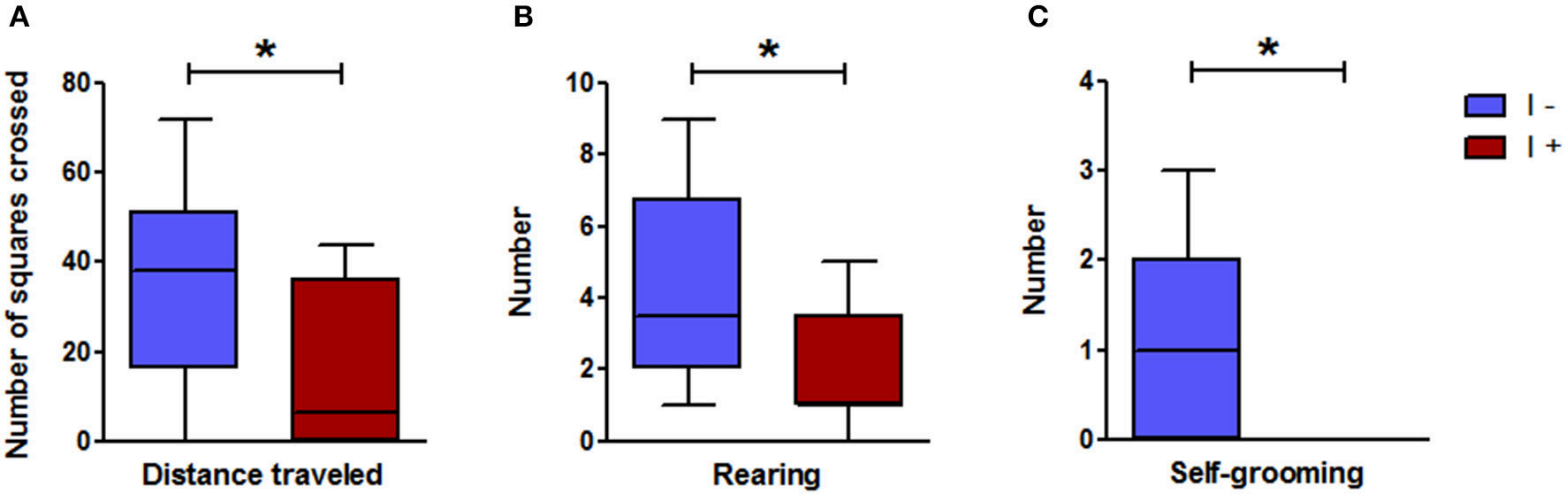
Open-field test in gnotobiotic rats mono-associated with the WT *E. coli* strain (“I+” rats) or with the mutant inactivated for the *tnaA* gene (“I−” rats). **(A)** Distance traveled, **(B)** Number of rearings, **(C)** Number of self-groomings (*n* = 12 rats/group, Mann-Whitney test). Data are medians with interquartile ranges. ^*^P < 0.05.

Despite an overall low level of exploration, behavioral differences were observed between the two groups in this test, which was carried out immediately after the elevated plus maze test. Of the 12 “I+” rats, 5 did not move from the corner in which they were originally placed, while this happened only in one “I−” rat. The distance traveled by the “I+” rats was significantly lower in these stressful conditions than that traveled by the “I−” rats, as was the number of rearings (Figure 9). Furthermore, all the “I+” rats for only 5 out of the 12 “I–” made no self-grooming at all (Figure 9). None of the rats visited the aversive center area, and no significant difference was observed between the 2 groups with regard to the latency time before leaving the initial corner [14(354.5) s in the “I+” rats and 6(11) s in the “I–” rats], and the number of defecations [1(1) in the “I+” and “I–” rats] (Mann-Whitney test; *n* = 12; *P* > 0.05).

#### Novelty test

**Figure 10:**
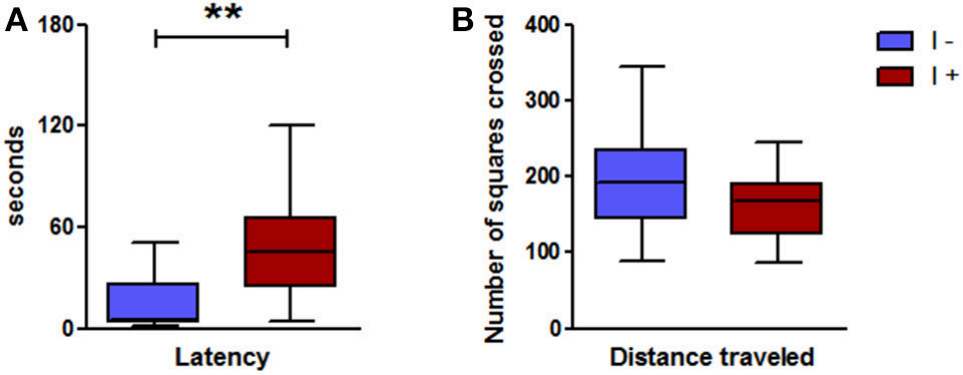
Novelty test and locomotor activity in gnotobiotic rats mono-associated with the WT *E. coli* strain (“I+” rats) or with the mutant inactivated for the *tnaA* gene (“I−” rats). **(A)** Latency to approach the object, **(B)** Distance traveled (*n* = 10–12 rats/group, Mann-Whitney test). Data are medians with interquartile ranges. ^**^P < 0.01.

This test was carried out in the second series of gnotobiotic rats (Figure 2) and confirmed the greater anxiety-like behavior of the “I+” rats. Indeed, the latency time, i.e., the time to go near to the object to explore it, was much longer in the “I+” rats than in the “I−” counterparts (Figure 10). This was not due to an impaired locomotor activity as the “I+” rats traveled the same distance as the “I−” rats (Figure 10). The cumulative time of exploration of the object over the 10-min observation was similar in the “I+” and “I−” rats, 70.1(42.2) s and 60.4(44.2) s, respectively (Mann-Whitney test; *n* = 10–12; *P* > 0.05).

#### Tail suspension test

**Figure 11:**
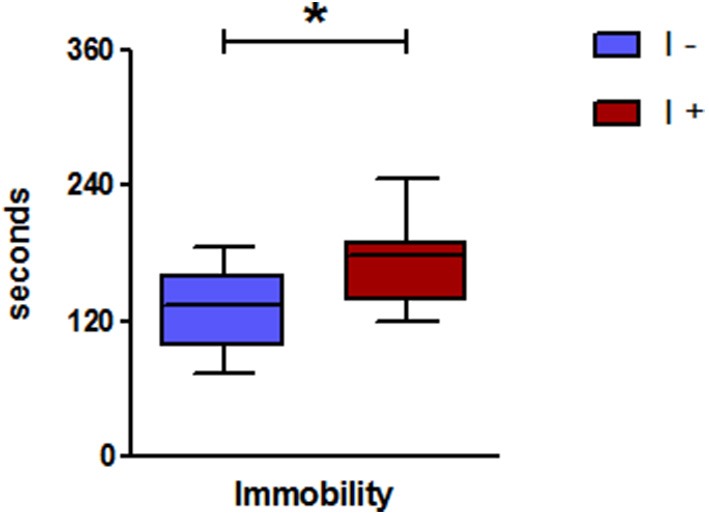
Tail suspension test in gnotobiotic rats mono-associated with the WT *E. coli* strain (“I+” rats) or with the mutant inactivated for the *tnaA* gene (“I−” rats). Duration of immobility (*n* = 11 rats/group, Mann-Whitney test). Data are medians with interquartile ranges. ^*^P < 0.05.

The “I+” rats showed a greater level of helplessness than the “I−” counterparts, as indicated by a longer immobility time (Figure 11).

### Memory performances were similar in the “I+” and “I−” rats

The object recognition task was performed in the second series of gnotobiotic rats, in the same arena as the novelty test, but 1 day later (Figure 2). During the 3-min sample phase, the exploration time of each of the two identical objects, A1 and A2, was similar in both groups of rats (data not shown). Then, during the 3-min test phase, when the rats were placed again, after a 1-h rest period, in the test arena, in the presence of a familiar object (A3) and a new object (B1), both groups spent significantly and identically more time exploring the new object. The exploration index (ratio of the time spent exploring the new object to the time spent exploring the two objects) was 61.3(45.1)% in the “I+” rats and 69.9(29.8)% in the “I−” rats (Mann-Whitney test; *n* = 10–12; *P* > 0.05). In addition, no significant difference occurred between the two groups regarding the total time of exploration of the objects, namely 15.5(25.1) s in the “I+” rats and 18.5(12.3) s in the “I−” rats (Mann-Whitney test; *n* = 10–12; *P* > 0.05).

### The “I+” rats had a greater eye blinking frequency than the “I−” rats

The eye blinking frequency was measured in the second series of gnotobiotic rats. During the 5-min measurement period, the “I+” rats had a significantly greater number of eye blinks than the “I−” rats, 2.80(2.15)/min vs. 1.80(1.20)/min, respectively (Mann-Whitney test; *n* = 11-12; *P* < 0.01).

### The “I+” rats and the “I−” rats had similar corticosterone concentrations in the plasma

As expected, the stress caused by the succession of the elevated plus maze and the open-field tests caused a dramatic increase of the corticosterone concentration in the plasma of both groups of rats (Kruskal-Wallis test followed by Dunn's multiple comparison test; *n* = 12; *P* < 0.001). Within the “I−” group, the non-stressed rats, which had not been subjected to the elevated plus maze and open-field tests (Figure 2), had a corticosterone concentration of 97(208) ng/mL plasma vs. 571(104) ng/mL plasma in the stressed counterparts (*P* < 0.001). In the same way, the non-stressed “I+” rats had a corticosterone concentration of 61(224) ng/mL plasma vs. 513(119) ng/mL plasma in the stressed counterparts (*P* < 0.01). There was no significant difference between the “I−” and “I+” groups, either in non-stressed or stressed state.

### Distribution of non-redundant *tna*A gene products in human microbiota

**Figure 12:**
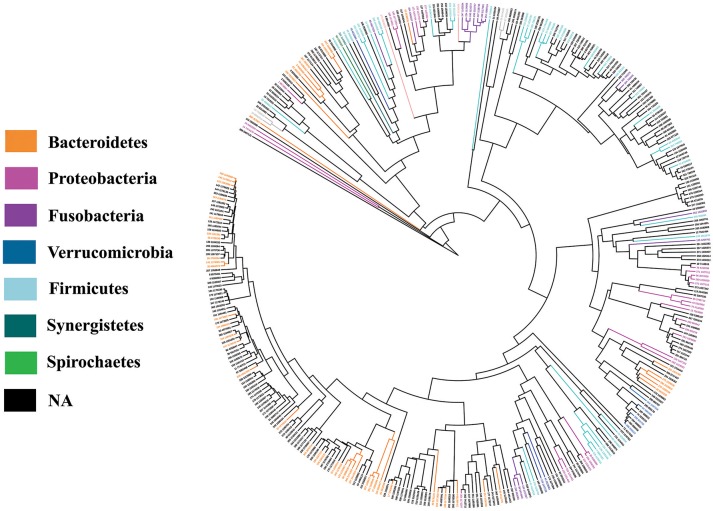
Phylogenetic analysis of TnaA proteins retrieved from the Human microbiota catalog. Assigned proteins are indicated in color according to the phyla they belong to, while the proteins that cannot be assigned to cultivated species are in black.

**Figure 13:**
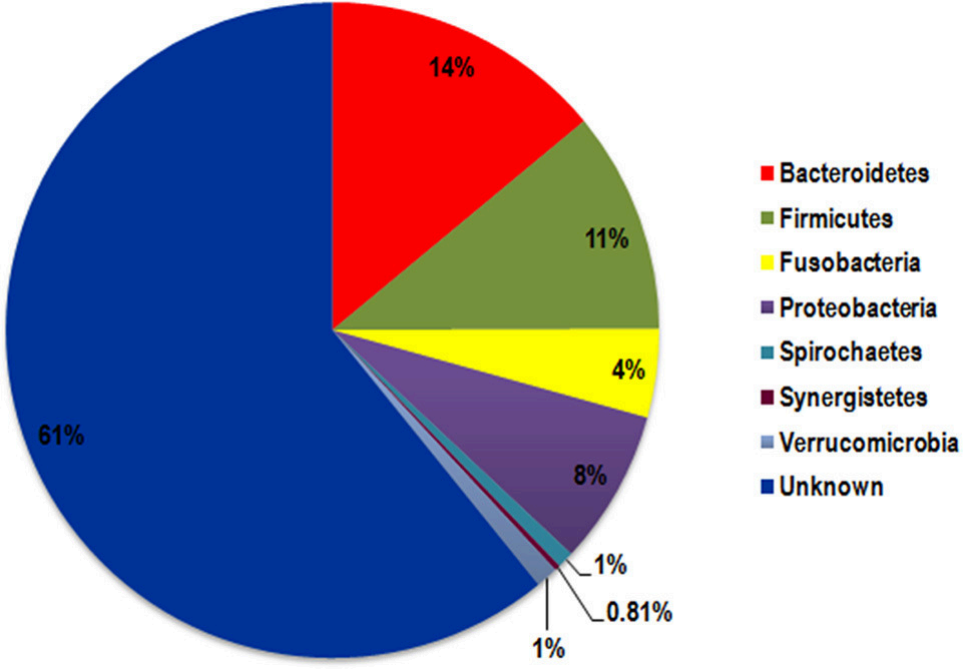
Distribution of the non-redundant TnaA proteins from the microbiota among the bacterial phyla. The pie-chart represents the relative percentage of TnaA-proteins retrieved from the Human microbiota catalog in the various bacterial phyla represented in the intestinal tract.

**Figure 14:**
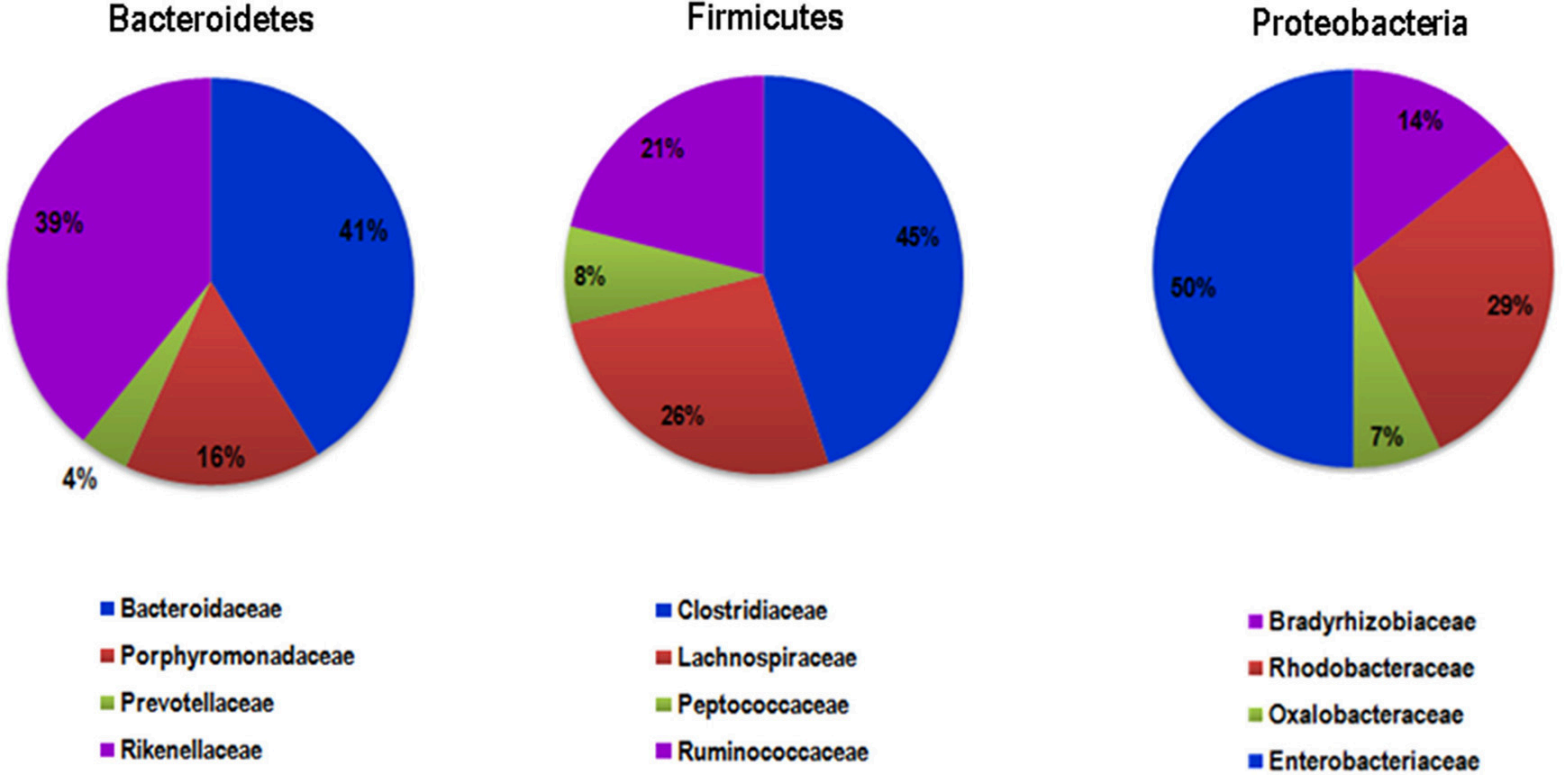
Pie charts showing the family-level distribution of TnaA for the 3 phyla containing the larger number of genes putatively encoding TnaA.

As we evidenced in this work that the microbiota TnaA activity affects the rat behavior, we investigated the distribution and richness of TnaA encoding genes in the human microbiota. The analysis of the human gut metagenomics catalog of ~10 million genes (Li et al., 2014), using a curated reference set of TnaA proteins, led to the identification of 373 bacterial genes putatively encoding for tryptophanases (i.e., *tna*A-like genes). Interestingly, the phylogenetic analysis of the gene products retrieved from the human microbiota catalog revealed that their majority (61%) originated from unknown species (Figures 12, 13). Taxonomic assignation of these sequences showed that they mainly belong to the *Firmicutes, Bacteroidetes* and *Proteobacteria* phyla (Figures 12, 13). Further study revealed that most of the assigned sequences derived from *Bacteroidaceae, Rikenellaceae, Clostridiaceae, Lachnospiraceae, Enterobacteriaceae*, and *Rhodobacteraceae* families (Figure 14). It is noteworthy that some of these bacterial families are known to be high producers of tryptophanase activity allowing the conversion of tryptophan into indole (Yoshida et al., 2009).

**Figure 15:**
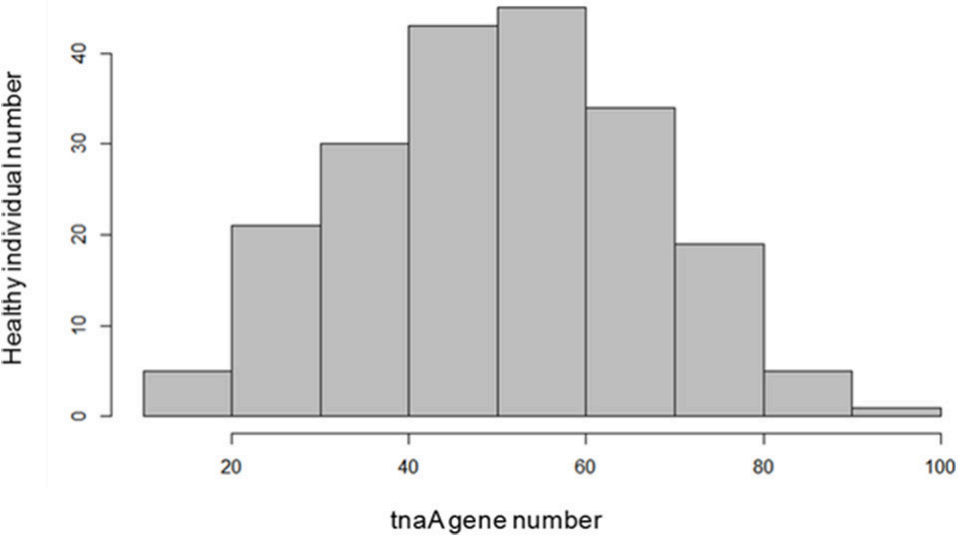
Richness of the non-redundant *tna*A genes within the microbiota of 203 healthy individuals.

The study of the *tna*A genes distribution within the microbiota of 203 healthy individuals showed that the richness of non-redundant *tna*A genes varied from 5 to 100. However, only a small number of healthy individuals exhibited below 20 (5 subjects) and above 80 (6 subjects) non redundant *tna*A genes in their microbiota (Figure 15).

## Discussion

Our results demonstrate that an overproduction of indole in the hindgut, whether it is acute and high, or whether it is chronic and moderate, is able to influence brain and behavior in rats.

We first confirmed that an acute systemic administration of oxindole or isatin had a depressant effect on the motor activity. The doses we injected were in the same order of magnitude as those used previously by Abel (1995) and Carpenedo et al. (1998) for isatin and oxindole, respectively. Although we used a different rat strain, we got the same results as them, i.e., a progressive decrease of the locomotor activity according to the drug dose. In addition to the locomotor activity, we measured the number of rearings. For both drugs, with the lower dose, this number was decreased to a greater extent than the locomotor activity, and was almost completely abolished with the higher dose. This could reflect a reduction of the muscular tone, a symptom which was reported by Abel (1995) and Carpenedo et al. (1998). As expected, virtually no oxindole or isatin could be detected in control rats' brains. With the higher dose of oxindole, namely 100 mg/kg, we measured in the brain a 5-fold greater concentration than the one reported by Carpenedo et al. (1998). This discrepancy may be due to the different delays between oxindole administration and brain collection, i.e., 2 h in the present study vs. 1 h in the study by Carpenedo et al. (1998). To the best of our knowledge, no kinetics study has been carried out on the accumulation of oxindole in the brain following systemic administration of this compound. It seems from our results that this accumulation can be greater than expected from previous findings. With regard to isatin, we do not have any reference from the literature since Abel (1995) did not measure isatin brain concentrations following systemic administration. In the rats treated with this compound, we found low levels of isatin, which were correlated to the injected dose. Surprisingly, appreciable amounts of oxindole were also detected in the brain of rats treated with isatin. This suggests that a metabolic pathway may lead from isatin to oxindole, although this has never been reported in the literature.

To show that indole increase in the digestive tract would increase brain levels of oxindole, Carpenedo et al. (1998) gave indole to rats *per os*. The gut microbiota, which is responsible for the metabolism of dietary tryptophan into indole, is the most abundant in the hindgut. Therefore, to mimic as well as possible the microbial production of indole in the gut, we chose to administer indole directly in the cecum of the rats. This route of administration allowed indole to be absorbed and further metabolized by the host, as ascertained by the presence of indoxylsulfate, the major final metabolite of indole, in urine. As hypothesized, oxindole, and isatin were also formed, as we detected them in plasma and brain. The dose of 500 mg/kg we injected was based on the work by Carpenedo et al. (1998) and on our results following systemic injection of oxindole and isatin. Interestingly, this dose led to oxindole and isatin brain levels roughly in the same order of magnitude as those observed with a systemic injection of 50 mg/kg of these drugs. The significant one-half reduction of the locomotor activity and the near elimination of the rearing behavior were also in the same order of magnitude. Thus, these findings show that an acute and high overproduction of indole in the gut can have a depressant effect on motor activity, possibly through the production of the neurodepressant compounds oxindole and isatin. However, our study reveals another pathway whereby indole could signal to the brain and induce behavior modifications. Indeed, a dramatic increase in the number of eye blinkings was observed in all indole-treated rats. Eye blinking increase was previously shown after a stimulation of the vagus nerve, in cats. This was ascribed to an activation of the oculomotor nucleus by afferent neurons from the DVC, the integrative center of autonomic functions that comprises cerebral nuclei of the vagus nerve (Valdés-Cruz et al., 2002). Therefore, we measured c-Fos expression in the DVC cells as an indicator of vagus nerve stimulation (Yang et al., 2004; Covasa and Ritter, 2005). c-Fos was overexpressed in indole-treated rats compared to control counterparts. This finding, together with the rapidity of the eye blinking onset (around 2 min after indole administration), and the fact that no eye blinking increase occurred in oxindole or isatin-treated rats, confirms that indole induced an activation of the vagal afferent fibers in the intestinal mucosa. However, at this stage, we do not know if this activation was due to indole itself, or to secondary signals whose production was triggered by indole. Interestingly, in a study on the beneficial effects of a probiotic treatment on brain functioning, it was shown that these effects were suppressed by a vagotomy. As a result, the authors concluded that the vagus nerve would be a major modulatory communication pathway between gut bacteria and the brain (Bravo et al., 2011). Butyrate, a short-chain fatty acid derived from microbial fermentation of dietary fibers, could be one of the bacterial signals involved in this pathway (Stilling et al., 2016). We show here that indole, a microbiota-derived amino acid metabolite, could be another effector.

We next investigated the situation of a chronic and moderate overproduction of indole in the hindgut. For this, we used gnotobiotic rats associated with a defined model microbiota. This kind of simplified model has already been used successfully by us and others, to demonstrate the effect of specific bacteria, enzymatic activities or metabolites on the host physiology (Stappenbeck et al., 2002; Sudo et al., 2004; Humblot et al., 2007; Marcobal et al., 2013). Here, we compared the effect of an overproduction of indole vs. no indole production on a set of behaviors. We found that the rats producing indole displayed consistently an enhanced anxiety-like behavior in several behavioral tests compared with rats producing no indole. This was striking in the elevated plus maze and the novelty tests, in which the key parameters reflecting an anxiety-like behavior, namely the visits to the open arms in the elevated plus maze and the latency time to visit the object in the novelty test, were clearly decreased and increased, respectively, in the “I+” rats. In the open-field test, interpretation of the results is less obvious. Indeed, none of the rats visited the center and the latency time before leaving the initial corner was rather long, and similar in both “I+” and “I−” rats. This may reflect a high level of anxiety in all rats, which may result from the fact that the open-field test was carried out immediately after the elevated plus maze test. Nevertheless, the distance traveled and the number of rearings in “I+” rats were significantly lower than in “I−” rats. In the open-field stressful conditions we used, such lower performances may rather reflect a greater anxiety than a decrease in general activity. The fact that these behaviors were not reduced in “I+” rats in any of the other behavioral tests reinforces this interpretation. In addition, other authors have reported that a decrease of locomotor and rearing activities in an open-field test can be corrected by the administration of an anxiolytic drug (Bhattacharya et al., 1991). In view of their greater anxiety-like behavior, it may seem surprising that, in the social interaction test, “I+” rats spent more time interacting with their counterparts than “I−” rats. However, dissociation between the behavioral responses in an open-field or elevated plus maze test and those obtained in a social interaction test have been previously described (Cecchi et al., 2002; Lee et al., 2008). In the same line, Ramos et al. (1997) showed that rats with longer social interaction times spent less time in the open arms of an elevated plus-maze. Eventually, the production of indole led to an increased immobility in the tail suspension test, but did not affect the memory performances as assessed by the object recognition task. Thus, overall, rats overproducing indole were characterized by greater anxiety-like and helplessness behaviors, compared with rats not producing indole. These findings are consistent with previous reports on the behavioral effects of isatin (Bhattacharya et al., 1991; Abel, 1995). Consequently, we looked for the presence of this compound in the brain of “I+” rats vs. “I−” rats. Actually, we could not detect isatin in any of the rats' brains and found traces of oxindole in a few of them only. Therefore, it seems unlikely that isatin, or oxindole, were responsible for the behavioral impairments observed in rats overproducing indole. Nevertheless, the measurements were made on the entire brain. Perhaps, a study focused on areas known as major binding sites of isatin, namely the cortex, the hypothalamus or the hippocampus (Crumeyrolle-Arias et al., 2003), could have allowed a better detection as well as evidenced concentration differences. Other derivatives, which remain to be identified, may also be involved. Indeed, the fact that substantial amounts of indoxylsulfate were present in the urine of “I+” rats, whereas none was detected in the urine of “I–,” proves that indole produced by the WT *E. coli* cells in the hindgut was absorbed and further metabolized. In this regard, it was shown very recently that some microbial metabolites of tryptophane, including indoxylsulfate, were able to activate the transcription factor aryl hydrocarbon receptor in astrocytes, resulting in a modulation of cerebral inflammation (Rothhammer et al., 2016). Alternatively, indole may have signaled to the brain via the vagus nerve, as the eye blinking frequency in “I+” rats, though much lower than the one observed following the acute administration of indole, was significantly higher than in “I−” rats. One can also hypothesize that an excessive microbial catabolism of tryptophan, by “trapping” this amino acid, could disrupt the host synthesis of tryptophan derivatives fundamental in brain functioning, i.e., serotonin and kynurenin. Tryptophan is an essential amino acid and precursor of the serotonin monoaminergic neurotransmitter, and it is now known that gut microbiota can impinge on tryptophan metabolic pathway leading to serotonin. Indeed, the level of peripheral serotonin is decreased in germ-free mice (Wikoff et al., 2009), and several groups, including ours, showed that the level and turn-over rate of brain serotonin is enhanced in germ-free mice and rats, compared to conventional ones (Clarke et al., 2013; Nishino et al., 2013; Crumeyrolle-Arias et al., 2014). Effect of gut microbiota on tryptophan metabolism along the kynurenin pathway is also possible, as the kynurenin/tryptophan plasma ratio is decreased in germ-free mice, and restored to control values by gut microbial colonization (O'Mahony et al., 2015). Overall, it appears that the influence of gut microbiota on tryptophan metabolism is complex and may therefore have multiple, favorable and unfavorable, consequences on brain and behavior.

Comparisons of germ-free and conventional rodents have consistently shown an exacerbated reactivity of the hypothalamic-pituitary-adrenal (HPA) axis in germ-free conditions, highlighting the pivotal role of the gut microbiota in the maturation and regulation of this axis (Sudo et al., 2004; Clarke et al., 2013; Crumeyrolle-Arias et al., 2014),. We thus examined if indole affected the systemic corticosterone concentration. There was no difference between the “I+” and “I−” groups, either at baseline or after subjecting the rats to stressful behavioral tests. It must be noticed that the post-stress plasma corticosterone concentrations were very high, comparable to those we had previously observed in germ-free rats (Crumeyrolle-Arias et al., 2014). Thus, it is possible that the colonization of the germ-free rats with a defined model microbiota consisting in *E. coli* cells only was not sufficient to restore a normal reactivity of the HPA axis. In this regard, Sudo et al. (2004) compared the systemic corticosterone elevation in response to stress in germ-free mice colonized at birth with an *E. coli* strain or with a *Bifidobacterium infantis* strain. The HPA axis reactivity of the mice mono-associated with *E. coli* was similar to germ-free mice, while mono-association with *B. infantis* restored a normal reactivity, similar to conventional mice. Thus, our results seem to confirm that *E. coli* is unable to promote a normal HPA axis maturation. This phenomenon may have masked a potential increasing effect of indole on the HPA axis reactivity, as could be expected in view of its anxiogenic effect. However, an absence of correlation between anxiety and depressive-like behaviors and elevated corticosterone level following a stress was already reported in the literature: in anxiety tests (Keck et al., 2003; Armario et al., 2012), as well as in experimental models of depression (Wigger and Neumann, 1999; Slotten et al., 2006). Therefore, in the present study, it is likely that the anxiety-like and depressive-like behaviors in “I+” rats originated in the disturbance of other(s) system(s) such as the cortico-limbic system, but this remains to be investigated.

The results obtained with the model of chronic and moderate overproduction of indole suggest that individuals whose intestinal microbiota is highly prone to produce indole could be more likely to develop anxiety or depressive disorders than those whose microbiota is less prone to produce indole. However, as it is not known to which extent the gut microbiota indole production potential can vary between individuals, we undertook an *in silico* analysis of metagenomic data focused on the *tna*A gene products responsible for the tryptophan to indole catabolism. This study shows for the first time that the potential of the microbiota to produce indole is very heterogeneous among healthy human subjects, given the high inter-individual amplitude of variation of the non-redundant *tna*A genes. Moreover, this study also shows that only a few people carry a potentially high-indole producing gut microbiota or, on the opposite, a potentially low-indole producing gut microbiota. In recent years, many studies pointed out that the gut microbiota could be involved in the pathophysiology of mood disorders. Notably, gut microbiota composition differences were found between healthy people and patients with major depressive disorder (MDD) (Naseribafrouei et al., 2014; Jiang et al., 2015; Kelly et al., 2016; Zheng et al., 2016). In addition, fecal transplantation experiments involving colonization of rodents either (i) with feces from irritable bowel syndrome patients with anxiety co-morbidity, led to anxiety in mice (De Palma et al., 2017), or (ii) with pooled feces from various MDD patients, led to anhedonia to sucrose (Kelly et al., 2016) or to helplessness (Zheng et al., 2016) in the recipient animals. In view of our findings, it would be interesting in the future to determine the proportion of indole-producing bacteria in such microbiota and to verify if a microbiota of the high indole-producer type predisposes to anxious or depressed mood, or is preferentially found in patients suffering from anxiety or MDD.

Overall, we have demonstrated that an acute and high overproduction of indole on one hand, and a chronic and moderate overproduction of indole on the other hand, result in distinct behavioral changes: a dramatic decrease of motor activity in the acute condition, and anxiety- and depressive-like behaviors in the chronic condition. Putative ways of indole action were identified conclusively in the model of acute overproduction—activation of vagal afferences, increased brain levels of oxindole and isatin—and still remain in the form of tracks in the model of chronic overproduction—possible increase in brain levels of oxindole as suggested by the detection of this molecule in some “I+” rats, possible activation of the vagus nerve as indicated by the increased eye blinking frequency in these rats. In the future, we aim to distinguish, in each model, the part of one or the other way of action in the development of the observed behaviors.

These findings are a first step toward the identification of the chemical mediators involved in the crosstalk between gut microbiota and brain. They are contributing, in association with other studies, to the development of a base of knowledge on these mediators. This is a prerequisite to design, on a rational basis, new and innovative therapies for the world-wide increasing neuropsychiatric diseases.

## Author contributions

MJ, MR, VD, EM, LN, and SR: Conceived the scientific ideas and designed the studies; MJ, AB, VD, LN, and SR: Conducted the animal studies and analyzed the behavioral tests; MJ, CP, BG, LN, and SR: Performed the laboratory analyses; MR, NP, and EM: Performed the bioinformatics analyses. MJ, MR, EM, CP, LN, and SR: Wrote the manuscript. All authors reviewed the manuscript and provided critical feedbacks.

### Conflict of interest statement

The authors declare that the research was conducted in the absence of any commercial or financial relationships that could be construed as a potential conflict of interest.

## Abbreviations

BHI: Brain Heart Infusion
CFU: Colony Forming Unit
DM: Dry Matter
DMSO: Dimethylsulfoxide
DVC: Dorsal Vagal Complex
HPA: Hypothalamic Pituitary Adrenal
MDD: Major Depressive Disorder
WT: Wild-Type.
